# A Novel Design of Temporomandibular Joint Prosthesis for Lateral Pterygoid Muscle Attachment: A Preliminary Study

**DOI:** 10.3389/fbioe.2020.630983

**Published:** 2021-01-21

**Authors:** Luxiang Zou, Yingqian Zhong, Yinze Xiong, Dongmei He, Xiang Li, Chuan Lu, Huimin Zhu

**Affiliations:** ^1^Department of Oral Surgery, Ninth People's Hospital, Shanghai Jiao Tong University School of Medicine, Shanghai, China; ^2^Shanghai Key Laboratory of Stomatology & Shanghai Research Institute of Stomatology, Shanghai, China; ^3^National Clinical Research Center of Stomatolog, Shanghai, China; ^4^State Key Laboratory of Mechanical System and Vibration, School of Mechanical Engineering, Shanghai Jiao Tong University, Shanghai, China

**Keywords:** temporomandibular joint, prosthesis, lateral pterygoid muscle, porous titanium scaffold, muscle attachment

## Abstract

**Introduction:** In temporomandibular joint (TMJ) replacement operation, due to the condylectomy, the lateral pterygoid muscle (LPM) lost attachment and had impact on the mandible kinematic function. This study aimed to design a novel TMJ replacement prosthesis for LPM attachment and to verify its feasibility by preliminary *in vitro* and *in vivo* experiments.

**Materials and Methods:** An artificial TMJ prosthesis designed with a porous structure on the condylar neck region for LPM attachment was fabricated by a 3D printed titanium (Ti) alloy. A rat myoblast cell line (L6) was tested for adhesion and biocompatibility with porous titanium scaffolds *in vitro* by cell counting Kit-8 (CCK-8), scanning electron microscope (SEM), flow cytometry (FCM), real-time quantitative polymerase chain reaction (RT-qPCR), immunocytofluorescense, western blotting, etc. The porous titanium scaffolds were further embedded in the rat intervertebral muscle to analyze muscle growth and biomechanical strength *in vivo*. The novel artificial TMJ prosthesis was implanted to reconstruct the goat's condyle and LPM reattachment was analyzed by hard tissue section and avulsion force test.

**Results:** L6 muscle cells showed good proliferation potential on the porous Ti scaffold under SEM scanning and FCM test. In RT-qPCR, immunocytofluorescense and western blotting tests, the L6 cell lines had good myogenic capacity when cultured on the scaffold with high expression of factors such as Myod1 and myoglobin, etc. In the *in vivo* experiment, muscles penetrated into the porous scaffold in both rats and goats. In rat's intervertebral muscle implantation, the avulsion force was 0.716 N/mm^2^ in 4 weeks after operation and was significantly increased to 0.801 N/mm^2^ at 8 weeks (*p* < 0.05). In goat condylar reconstruction with the porous scaffold prosthesis, muscles attached to the prosthesis with the avulsion force of 0.436 N/mm^2^ at 8 weeks, but was smaller than the biological muscle-bone attachment force.

**Conclusion:** The novel designed TMJ prosthesis can help LPM attach to its porous titanium scaffold structure area for future function.

## Introduction

The temporomandibular joint (TMJ) plays a key role in mouth opening, speech, chewing, and swallowing. TMJ disease is common in clinical settings. At present, artificial total joint replacement has become a mainstay treatment modality for advanced osteoarthrosis, condylar tumors, TMJ ankylosis, autologous bone graft failure, and other conditions (Mercuri et al., 2007; Guarda-Nardini et al., 2008). However, removal of the condyle also leads to the detachment of the lateral pterygoid muscle (LPM) which participates in the movement of mandibular laterotrusion and protrusion (Westermark, 2010; Celebi et al., 2011; Zheng et al., 2019). Although it has been reported that muscle can reattach to an autologous bone graft (Wang et al., 2018), it has not been found to form a reattachment with the metal of the artificial joint. The loss of attachment of the pterygoid muscle results in lateral and forward movement limitation and serves as an obstacle in fine chewing functions. Studies have shown that the natural condylar translational movement range is about 16 mm, whereas the artificial joint can only make a rotation movement <6 mm (Sonnenburg and Sonnenburg, 1985; Mercuri et al., 1995; Wojczyńska et al., 2016). This increases the burden on other muscles and the contralateral natural joint, leading to discomfort and/or internal derangement of the contralateral joint (Ramos and Mesnard, 2015). Our previous study also found out that more than 30% of patients with unilateral joint replacement complained about discomfort in their contralateral joints, including clicking, soreness, muscle tension, etc. (Zou et al., 2019).

**Figure 1:**
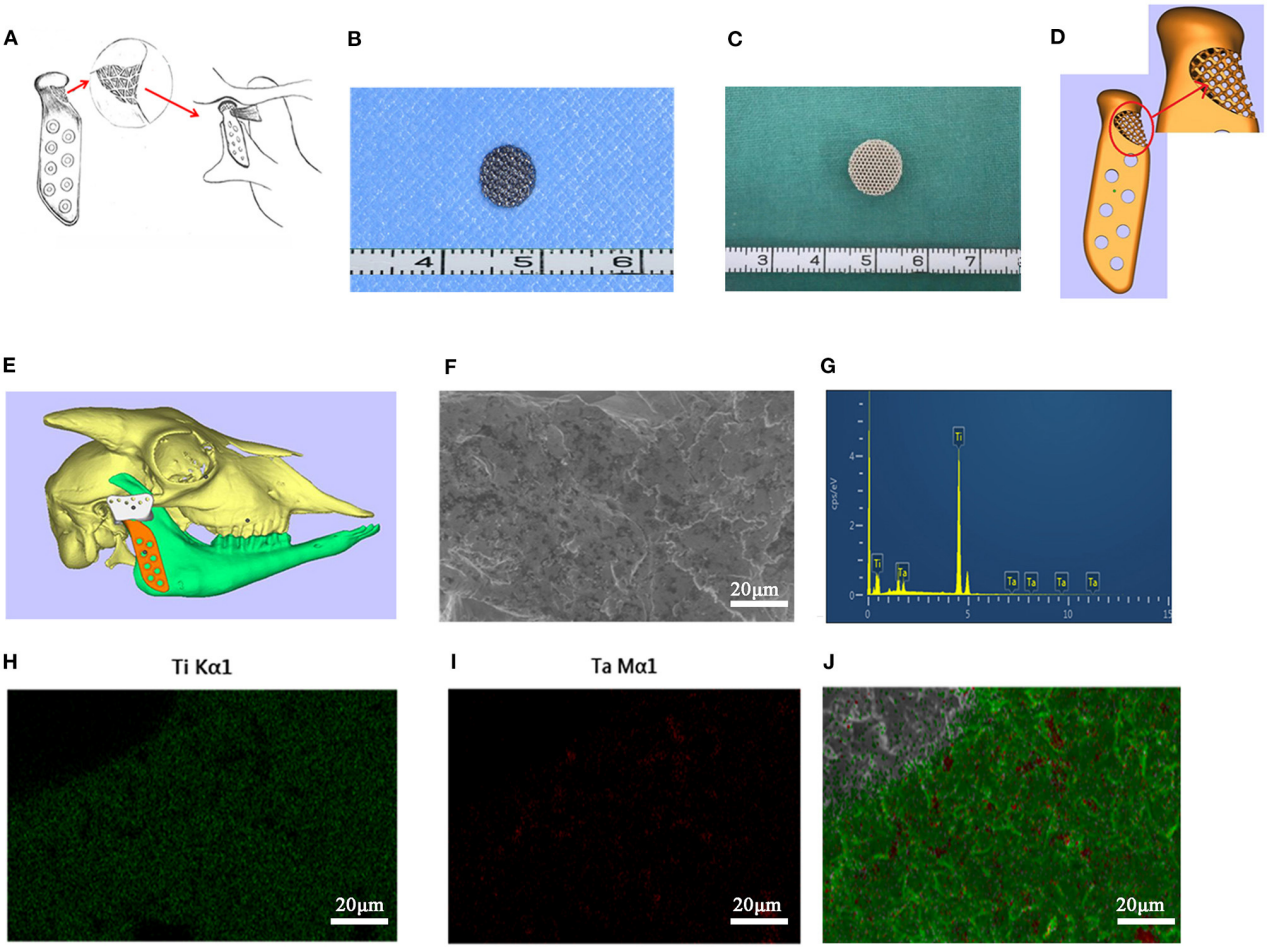
The 3D printed titanium porous scaffold and the elements analysis. **(A)** The schematic diagram of “LPM functional reattachment region” of TMJ prosthesis. **(B)** The 3D printed titanium scaffold for cytological experiments. **(C)** The 3D printed titanium scaffold for rat intervertebral implantation. **(D)** The CAD design of the novel prosthesis. **(E)** Simulated surgery and implantation of prosthesis in goat with CAD. **(F)** SEM scanning of cytological used Titanium scaffold. **(G)** EDS test of the elements in the 3D printed scaffolds. **(H,I)** Mapping analysis of the elements in the 3D printed scaffolds. **(J)** Merge of the EDS mapping analysis.

Based on the above, we propose a hypothesis that if the LPM can reattach to the artificial joint, it will promote the function of the joint (Figure 1A). In this study, we used 3D printing technology to establish a porous structure in the TMJ prosthesis for LPM reattachment. We then tested its feasibility through an *in vitro* cell experiment and *in vivo* in rat intramuscular implantation and goat condyle reconstruction to test its biocompatibility.

## Materials and Methods

This study was approved by the local ethics board of the hospital (Jiuyuan Lunshen, No. 73, 2015).

### Design and Manufacture of 3D Printed Porous Titanium Scaffold and TMJ Prosthesis

A porous titanium structure with a diameter of 5 mm, a thickness of 2 mm, a pore diameter of 500 μm, and a porosity of 75% was designed for cell adhesion experiments *in vitro*. In addition, a porous titanium scaffold with a diameter of 10 mm, a thickness of 5 mm, and same pore diameter and porosity as the first structure was designed for the use of intervertebral muscles in the rat's back implantation. Based on the Computed Tomography (CT) dicom data of goat mandibles, a prosthesis for condyle replacement was designed with a porous structure in the neck for LPM reattachment (Figures 1B–E). The scanning electron microscopy (SEM) and energy dispersive spectrometer (EDS) scanning were used to confirm the element consist of the printed scaffold (Figures 1F–J). All the above scaffolds and prosthesis were produced using 3D printing by Ti6Al4V (Sunshine Co Ltd, Shanghai, China). Ultrasonic cleaning and disinfection under high temperature and high pressure conditions were also performed.

### Cytology Evaluation of 3D Printed Porous Titanium Scaffold for Myogenic Cell Adhesion and Bioactivity

The porous titanium scaffold materials were fully immersed in α-MEM medium containing 10% fetal bovine serum (FBS) and cultured at 37°C in a 5% CO_2_ incubator for 72 h. The material leaching liquid was collected and stored in a refrigerator at 4°C.

The L6 rat myoblast cell line (purchased from the Chinese Academy of Biology Cell Bank, Shanghai) was inoculated into a 96-well plate at a density of 2 × 10^3^ cells per well. The material leaching liquid and a 10% FBS α-MEM complete medium were also added. The observation period was every 24 h and lasted for 5 days, with five replicate wells set in each group. The proliferation of cells in the two groups was determined by a cell counting kit-8 (CCK-8) test in order to study the biological toxicity impact of porous titanium material on L6 cells.

The L6 cells were inoculated on the surface of the porous titanium scaffold at a concentration of 1 × 10 ^6^/ml and cultured in a 37°C 5% CO_2_ incubator for 4 h to allow the cells to adhere to the material. They were then cultured in a complete medium containing of 10% serum, which was changed every other day. After days 1, 3, and 5 of incubation, the cells were fixed with 2.5% glutaraldehyde for 4 h at room temperature, and the morphology, adhesion, as well as proliferation of the cells were observed by SEM.

Flow cytometric analysis was carried out to verify the apoptosis which was led by the L6 cells co-cultured with scaffolds due to impact of metal material after 1, 3, and 5 days. Furthermore, after co-culturing with scaffolds, the L6 cells were stained by Calcein-AM and propidium iodide (PI) to label living cells and dead cells, respectively. In the fluorescence scan, the Calcein-AM stained living cells showed green fluorescence and PI stained dead cells showed red fluorescence.

Total RNA was obtained from the L6 cells cultured on the scaffold for 3 days by using RNA Express Total RNA Kit (Ncmbio, Suzhou, China) according to the manufacturer's instructions. NanoDrop 2000/2000C spectrophotometer was used to tested the purity and concentration of RNA at wavelengths of 260/280 nm. PrimeScript™ RT Reagent Kit (TaKaRa Biotechnology) was then used to reverse transcribe 1 μg of extracted RNA into cDNA. The resultant cDNA was used as template in the TB Green® Premix ExTaqTM Kit (TaKaRa Biotechnology) master mix and real-time quantitative polymerase chain reaction (RT-qPCR) reactions was performed on the Light Cycler96 Real-Time PCR System (Roche. Ltd, Switzerland). And the rat primer sets used were displayed in Table 1.

**Table 1:** Primers for the target gene.

**Gene name**		**5'-3' sequence (forward; reverse)**
Integrin-β1	Forward	5'-CTGGTTCTATTTCACCTACTCAG-3'
	Reverse	5'-CCAGTAGGACAGTCTGGAG-3
VCL	Forward	5'-CTACAACTCCCATCAAGCTG-3'
	Reverse	5'-TCTCGTCAAATACCTCTTCCC-3'
Desmin	Forward	5'-TCTCAACTTCCGAGAAACCA-3'
	Reverse	5'-TCAATGGTCTTGATCATCACTG-3'
MYH4	Forward	5'-TCATCTGGTAACACAAGAGGTGC-3'
	Reverse	5'-ACTTCCGGAGGTAAGGAGCA-3'
MyoD1	Forward	5'-ATGGCATGATGGATTACAGC-3'
	Reverse	5'-GACGCCTCACTGTAGTAGG-3'
TNN1	Forward	5'-GATGGAGAAATTGAAGCAACAG-3'
	Reverse	5'-CCCTTTCGGAATTTCTGGG-3'
Myoglobin	Forward	5'-GCAGGCTCAAGAAAGTGAATGA-3'
	Reverse	5'-TAGGCGCTCAATGTACTGGAT-3'
GAPDH	Forward	5'-AACTCCCATTCTTCCACCT-3'
	Reverse	5'-TTGTCATACCAGGAAATGAGC-3'

After 3 days of co-cultured with scaffold incubation, the scaffold material combined with the cells was fixed with 4% paraformaldehyde and permeabilized with 0.5% Triton X-100 (Sigma Aldrich) for 10 min at room temperature. It was then blocked with 5% bovine serum albumin (BSA) for 30 min and incubated with the primary antibodies for the cell adhesion and myogenic-related proteins (integrin-β_1_, 1:200; myoD, 1:200; Desmin, 1:200, Myoglobin, 1:100) overnight at 4°C. After that, the secondary anti-bodies and the Fluorescein isothiocyanate-labeled (FITC) phalloidin for cytoskeleton staining were incubated for 1 h at 37°C. The results were tested using immunofluorescence detection to confirm adhesion and biological activity of the L6 myoblasts on the 3D printed porous titanium scaffolds.

To define the biological activity potential of the L6 myoblasts on the Titanium scaffold, total cellular proteins (TCPs) were extracted from both the L6 cells cultivated on the scaffold and the common culture dish to be used as a control, after 3 days of inoculation for western blotting. The membranes were kept in 5% skim milk in 1 × TBST (Tris-buffered saline with 0.1% Tween 20) at room temperature for 1 h and then incubated with the primary antibodies (Gapdh, HuaxingBio, 1:3000; Desmin, Zenbio, 1:1000; MyoD, HuaxingBio, 1:1000; Myoglobin, Zenbio, 1:1000; Integrin-β1, HuaxingBio, 1:2000, TNNT1, HuaxingBio, 1:1000; VCL, HuaxingBio, 1:1000) overnight shaking at 4°C. Thereafter, the secondary anti-bodies were incubated for 1 h at room temperature and the antibody reactivity was visualized by using Bio-rad Gel Doc XR+ image scanning (Bio-rad. Inc., CA, USA).

### Rat Intramuscular Implantation of 3D Printed Porous Titanium Scaffold for Muscle Attachment Evaluation

**Figure 2:**
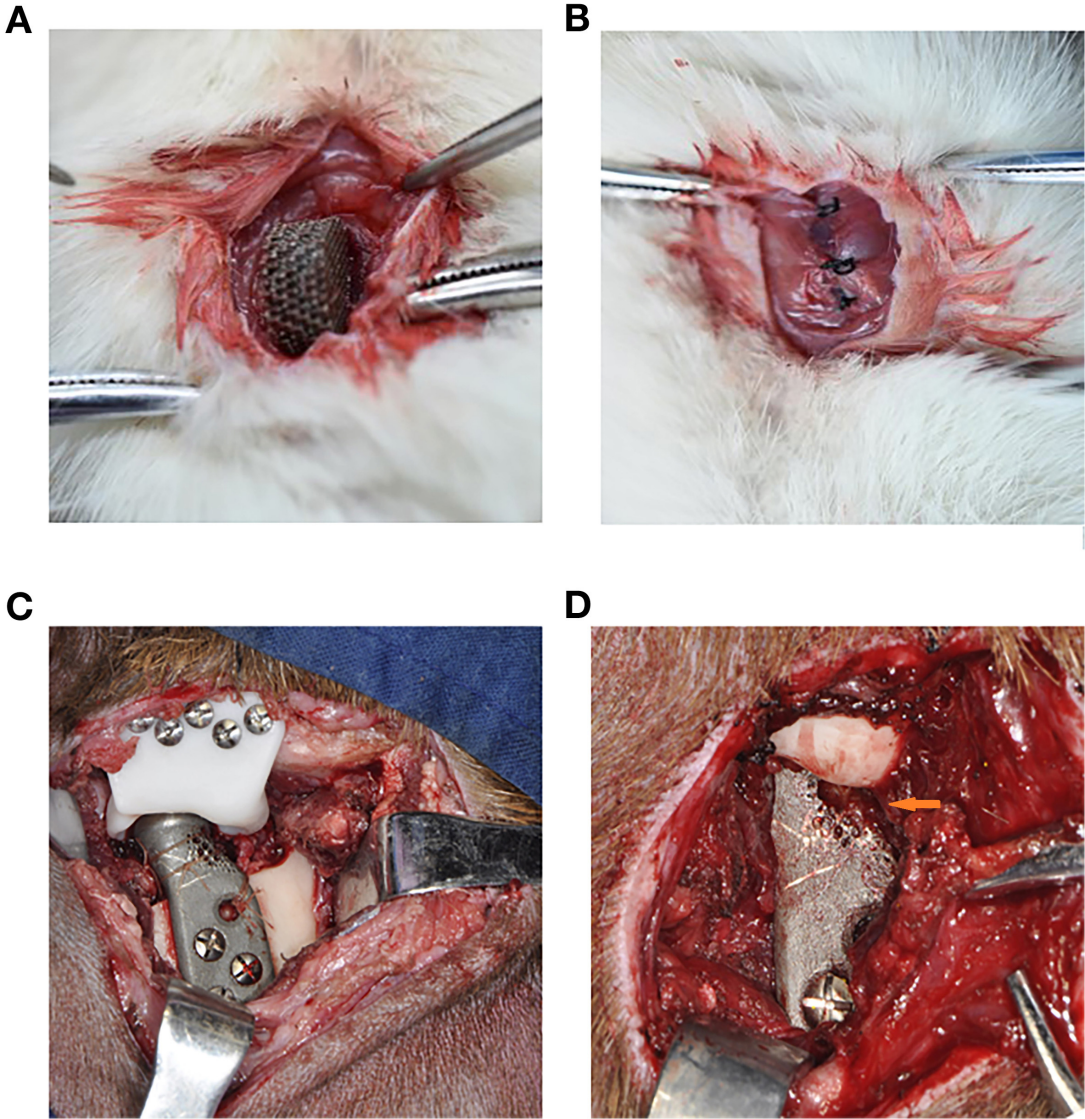
The surgical procedure of the *in vivo* experiment of rats and goats. **(A)** The implantation of the Titanium scaffold into the intervertebral muscle. **(B)** The suture, make the muscle surround the scaffold completely. **(C)** The implantation of the porous prosthesis and fasten the LPM with 5-0 absorbable suture to the prosthesis. **(D)** 8 weeks after surgery, the LPM grow into the porous prosthesis (Orange arrow).

After chloral hydrate injection anesthesia, the porous titanium scaffold with a diameter of 10 mm and a thickness of 5 mm was implanted into the intervertebral muscles of both sides of six male Sprague-Dawley rats (6 weeks), with one scaffold on each side (Figures 2A,B). The rats were then overdosed with an excess of anesthetic on weeks 4 and 8. Each time 3 rats were sacrificed and one side of the fresh samples were then tested for the maximum peeling off force of the porous titanium scaffold-muscle using a tensile tester (Instron 3345, Shanghai, China); while the other side specimens were fixed in 4% paraformaldehyde for 24 h and embedded in resin for hard tissue slicing. The distribution of muscle and collagen fibers was distinguished by Van Gieson (V-G) staining. The microscopy scans mainly focused on the integration of tissue-scaffold interface integration and angiogenesis in slice. Three fields from the upper, middle and lower part of the complete image acquisition were selected randomly for each sample, which were analyzed by Image J software (NIH, USA) to quantify the percentage of tissue ingrowth area of the total pore area and to demonstrate the integration of the muscle-metal interface as well as the muscle and collagen distribution.

### Goat Condylar Replacement by Novel Designed Prosthesis for LPM Attachment

Six male 12-month-old goats were used for right condyle removal and reconstruction with a novel designed prosthesis under general anesthesia (Figure 2C). Their left TMJ was untouched as a control method. Eight weeks after their operations, the goats were euthanized in order to enable mandible removal. Three of the prosthesis specimens was used to test the avulsion force of the LPM from the prosthesis with the tensile tester. The other three prostheses along with the LPM was fixed with 4% paraformaldehyde and prepared for hard tissue slicing with hematoxylin-eosin (HE) staining and V-G staining. The six normal condyles were resected with LPM attachment for avulsion force for use as a control base. The passive maximum incision opening (MIO) of the goats pre- and post-operation were also recorded as a biological function test of the prosthesis.

### Statistical Analysis

All statistical analyses were performed with SPSS software (version 19.0). The results of measurement were displayed as mean ± standard deviation (SD). The independent-sample *t*-test was used for RT-qPCR, western blotting measurement, histological analysis and avulsion force comparison, and one-way ANOVA test was used for CCK-8 proliferation rate. A *p-*value of 0.05 was considered statistically significant.

## Results

**Figure 3:**
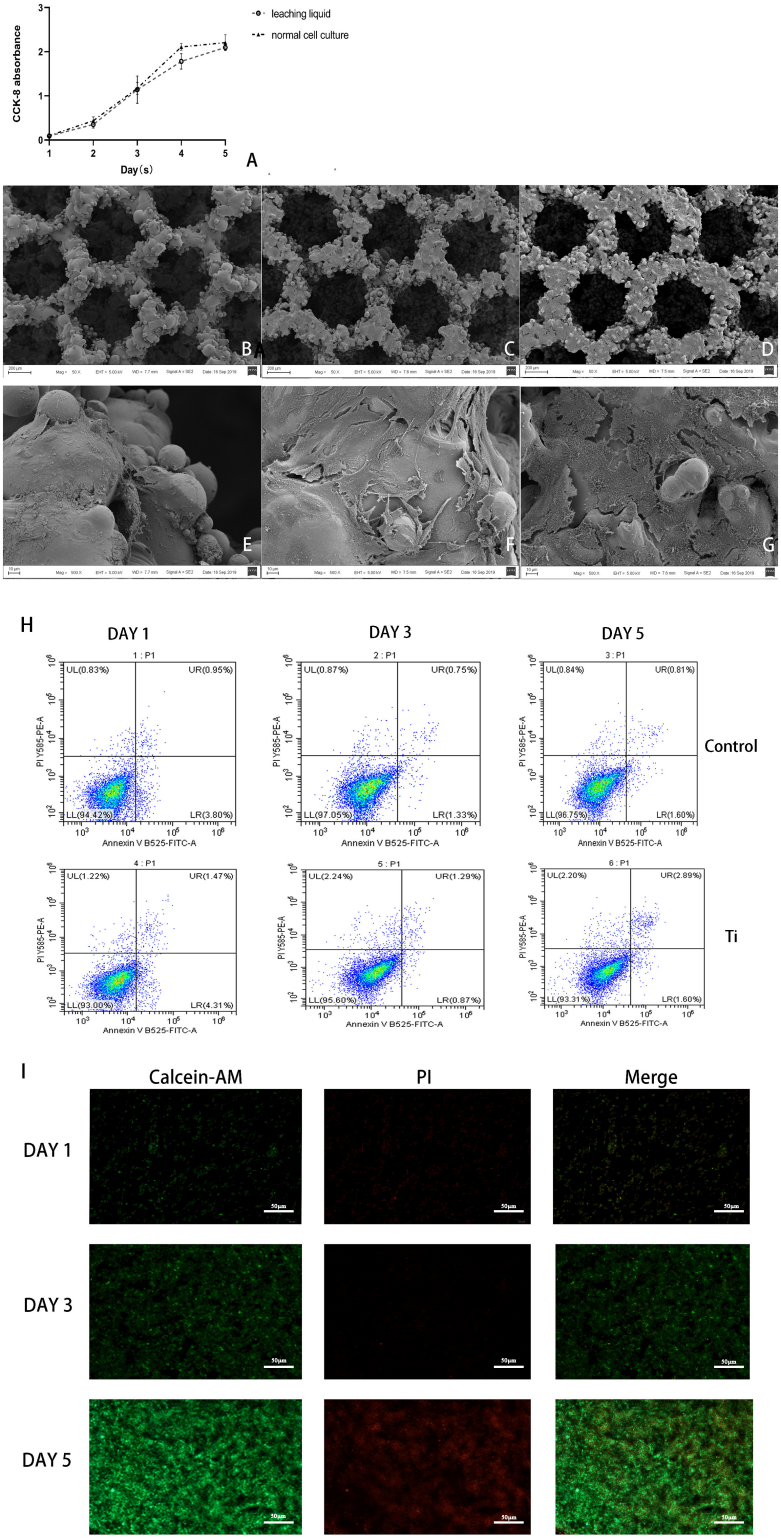
The biosafety of the scaffold were verified after co-cultivation with the scaffold. **(A)** CCK-8 test of cell proliferation in leaching liquid and ordinary culture medium, and in both situations, cells proliferate actively with no statistical difference (*p* > 0.05). **(B–G)** SEM scanning of L6 myoblast cultivated on the Titanium scaffold, **(B,E)** showed in 1 day, **(C,F)** showed in 3 days, **(D,G)** showed in 5 days, could see significant cell proliferation in 5 days, **(B–D)** 50 x, **(E–G)** 500 x. **(H)** Flow cytometry detection analysis of the cells cultured in dishes and on the Ti scaffold; and both the cells cultured in dishes or on the scaffold showed high activity with low apoptosis rate. **(I)** The cells on the scaffolds were taken by laser confocal microscopy (Green are living cells, and red are dead cells) in 1, 3, and 5 days. Significant proliferation with low cell death rate can be observed.

The results of CCK-8 cell proliferation test experiment showed that there was no significant difference in cell proliferation between the material leaching solution culture group and the normal medium group, indicating that the porous titanium material had no obvious toxicity to L6 cell proliferation (Figure 3A).

SEM observation showed that the L6 cells adhered tightly to the porous titanium scaffold material, with continuous cell proliferation and the number of cells increasing significantly. Under the 50-fold microscope, a large number of cells can be seen sticking to the scaffold material. Furthermore, under the 500-fold microscope, the cells can be seen to be stretched, and reveal pseudopods. The pseudopods allow the cells to connect to each other and across the pores of the material, which can be seen from adjacent cells. Moreover, some cells even merged to form a sheet, and a large amount of secretory matrix is visible covering the surface of the material (Figures 3B–G). Flow cytometric analysis of L6 co-cultured with scaffolds revealed that the co-cultured on the scaffold did not cause an obvious increase in apoptosis due to the metal iron release or scaffold structure compared with dishes (Figure 3H). Moreover, after co-culturing with scaffolds, the Calcein-AM and PI staining results revealed that the L6 cells exhibited good biocompatibility and proliferation capacity cultured on the scaffold (Figure 3I).

**Figure 4:**
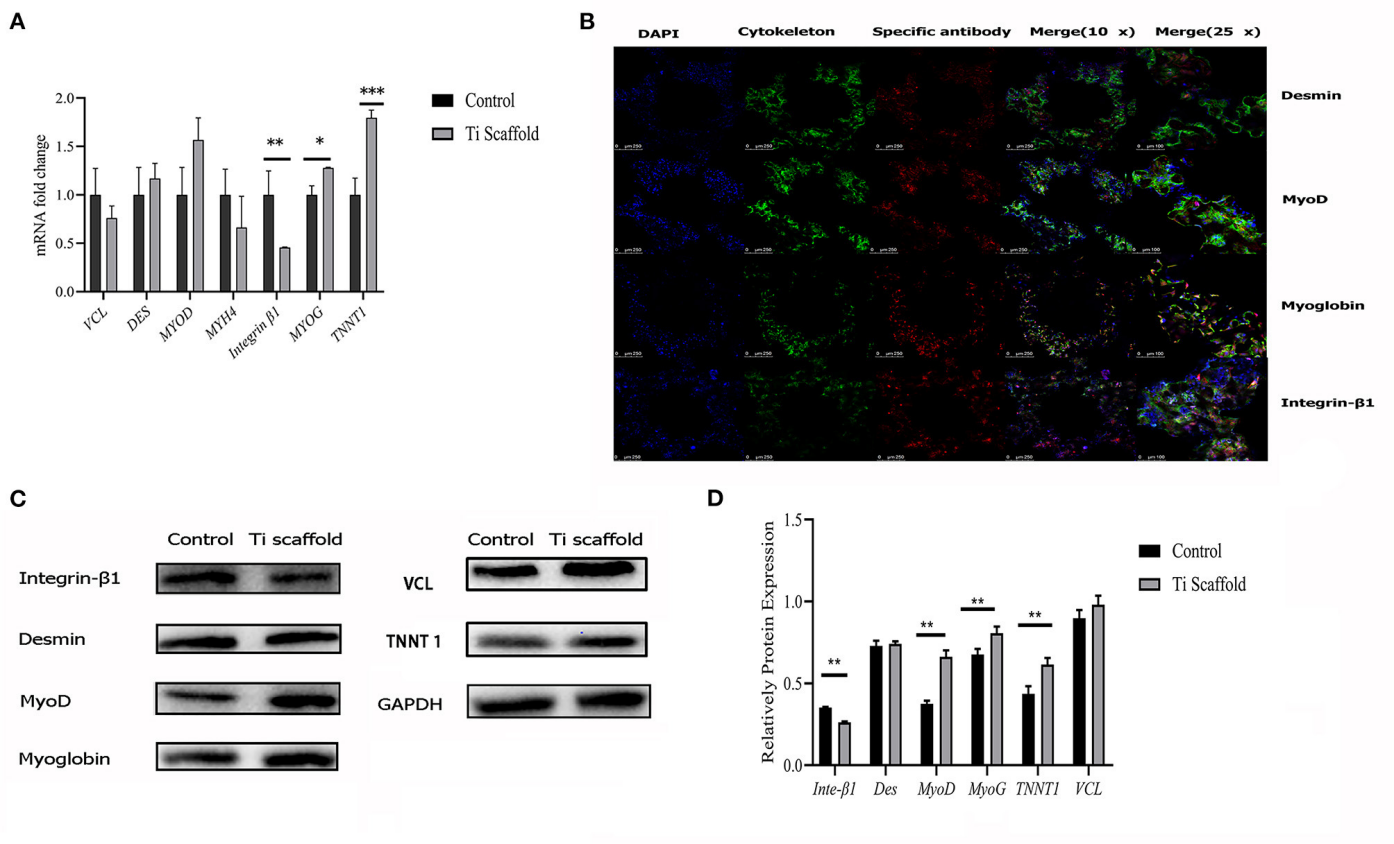
Bioactivity test of L6 myoblast on Titanium scaffold *in vitro*. **(A)** RT-qPCR analysis of mRNA expression fold change of integrin-β_1_, Desmin, MyoD, Myoglobin, TNNT1 and VCL expression in dish cultured and scaffold incubated group. The data are presented as the mean ± SD (*p < 0.05, **p < 0.01, ***p < 0.001). **(B)** Confocal microscopy with immunofluorescence of nuclei, cytoskeleton, Desmin, integrin-β1, MyoD and Myoglobin. **(C)** Western blot results of integrin-β_1_, Desmin, MyoD, Myoglobin, TNNT1, and VCL protein expression in dish cultured and scaffold incubated group. **(D)** Proteins expression levels were normalized to the expression of GAPDH. The data are presented as the mean ± SD (**p < 0.01).

The RT-qPCR results showed that when co-cultured on the scaffold, the L6 cells showed even stronger myogenic bioactivity compared with cultured in the dishes (Figure 4A). The results of confocal microscopy scanning showed that the FITC labeled cytoskeleton and integrin-β1 were abundantly expressed after the L6 cells were co-cultured with porous titanium scaffolds. The myogenic-related factors as MyoD, Myoglobin, Desmin were significantly expressed, indicating that the myoblasts and porous titanium scaffold have strong biocompatibility *in vitro* (Figure 4B). The western blot results of cell culture on scaffold and the control group showed that the L6 myoblast showed greater myogenic differentiation factor expression on the 3D printed titanium scaffold, and the expression of MyoD, myoglobin and TNNT1 were significantly higher than the control group, with no difference in Desmin. However, the expression of integrin-β_1_ factor in the scaffold group was lower than that in control group (*p* < 0.05, Figures 4C,D).

**Figure 5:**
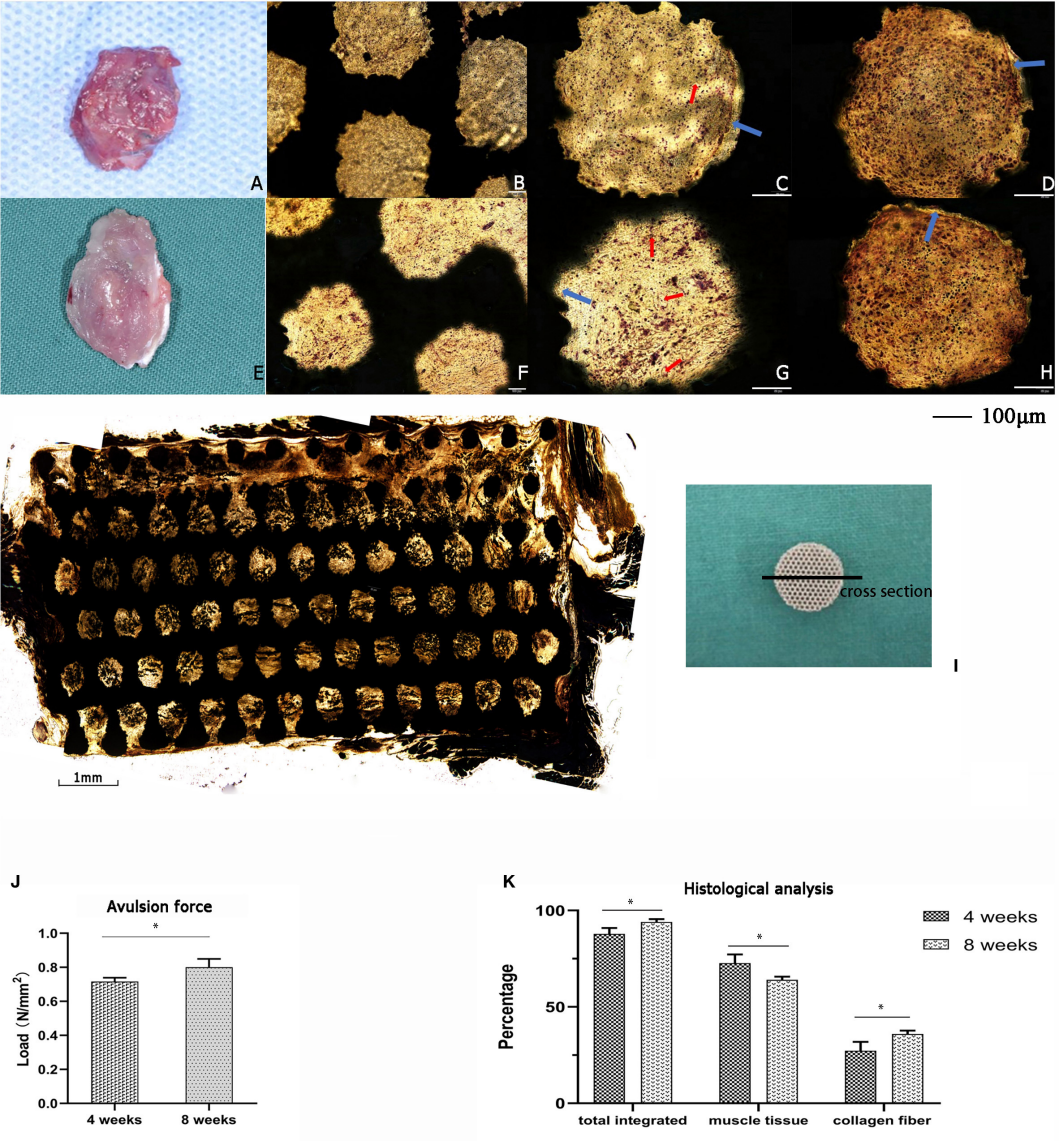
The implantation of titanium scaffold into the intervertebral muscle of rats. **(A–D)** The specimen of 4 weeks, **(A)** Gross specimen, **(B)** hard slicing with V-G staining, 10 x, **(C,D)** hard slicing with V-G staining, 20 x; **(E–H)** The specimen of 8 weeks, **(E)** Gross specimen, **(F)** hard slicing with V-G staining, 10 x, **(G,H)** hard slicing with V-G staining, 20 x. Blue arrow: the blank gap between tissue and pore, red arrow: the newly formed capillary. **(I)** Histologic cross-section at 8 weeks showing the ingrowth of muscle tissue into the scaffold,4 x. **(J)** The avulsion force of titanium scaffold implanted in rats for 4 and 8 weeks; with a significant increase in 8 weeks (*p < 0.05). **(K)** The histological analysis of the tissue integrated percentage and distribution in rats. The data are presented as the mean ± SD (*p < 0.05).

For the *in vivo* experiment, the rat intervertebral muscle porous titanium scaffold was implanted for 4 and 8 week-periods. After drawing materials, the gross overview showed that the muscles and materials of the two groups were both well-combined, the color was red-pink and soft to the touch, with no ectopic bone formation (Figures 5A,E). Additionally, the V-G staining of the slice showed that the muscle cells and collagen fibers grew into the porous titanium scaffold together, and some porous titanium scaffolds had blank areas at the edges of the scaffold and the tissue, which means that they were not fully integrated. The newly formed red stained tube-like structure is visible in the pores, which is more common in the week 8 samples and demonstrates a new capillary (Figures 5B–D,F–H). The muscle tissue can grow into majority of the pores of in the cross-section view of the specimen of 8 week, but the distribution was not uniform (Figure 5I). The avulsion force for the 4 weeks group was 0.716 N/mm^2^ and increased to 0.801 N/mm^2^ at 8 weeks (Figure 5J). And in the histological analysis, the average rate of integration was 88.36% at 4 weeks, which further increased to 93.27% (*p* < 0.05) at 8 weeks, at which the proportion of collagen fibers increased from 27.37% to 35.98% (*p* < 0.05) and the proportion of muscle fibers decreased accordingly (Figure 5K).

**Figure 6:**
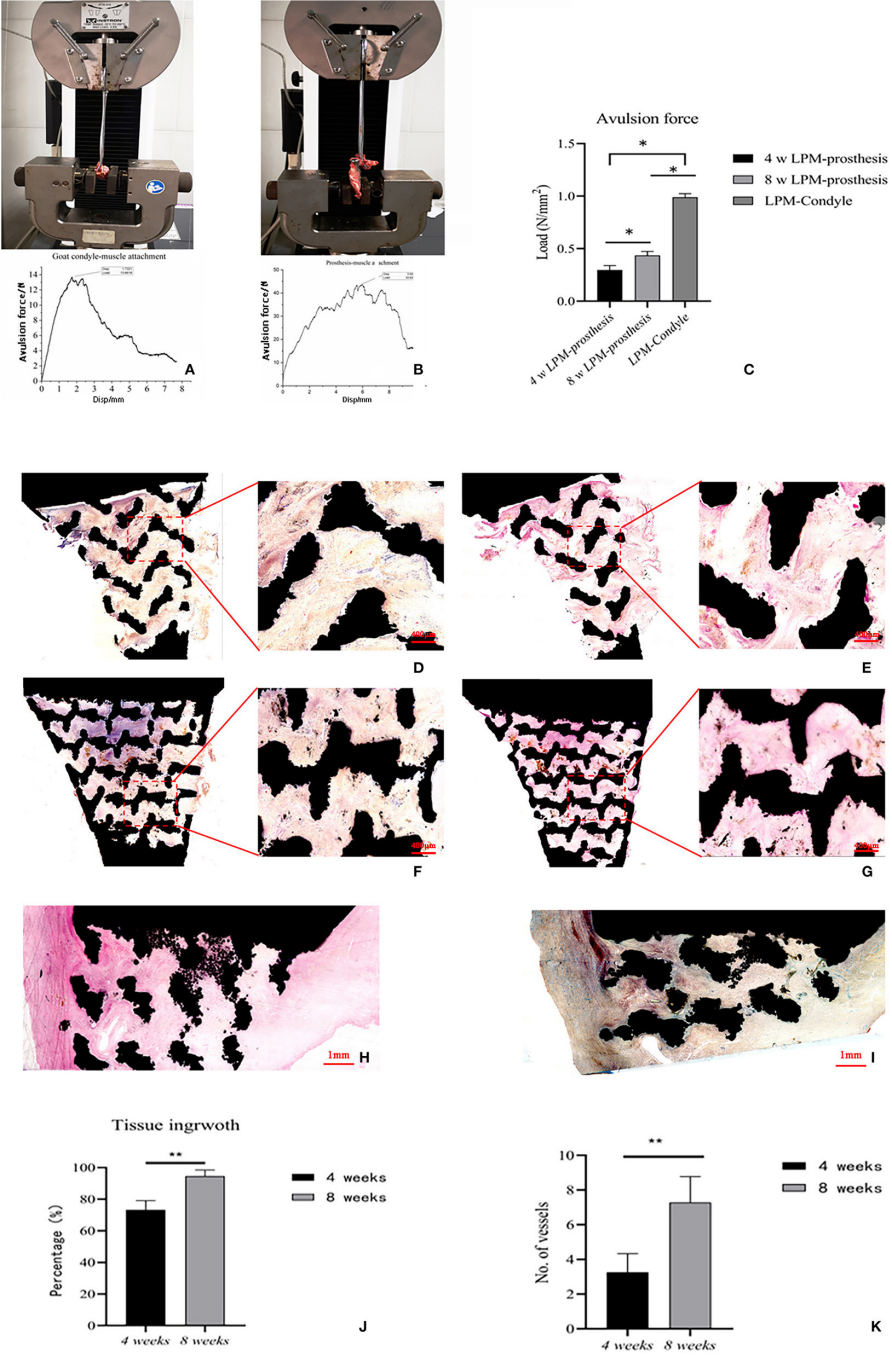
The *in-situ* reconstruction of goat temporomandibular joint with porous Titanium prosthesis. **(A)** The avulsion force test of goat condyle-LPM. **(B)** The avulsion force test of prosthesis-LPM reattachment. **(C)** The average maximum avulsion pressure of condyle-LPM and prosthesis-LPM in both 4 and 8 weeks. The data are presented as the mean ± SD (*p < 0.05). **(D–I)** The hard slicing of prosthesis-LPM specimen. **(D)** The V-G staining of hard slice of muscle-reattachment functional prosthesis at 4 weeks, 4x−10x. **(E)** The HE staining of hard slice of muscle-reattachment functional prosthesis at 4 weeks, 4x−10x. **(F)** The V-G staining of hard slice of muscle-reattachment functional prosthesis at 8 weeks, 4x−10x. **(G)** The HE staining of hard slice of muscle-reattachment functional prosthesis at 8 weeks, higher percentage of space were occupied with new ingrowth tissue with vessels compared with 4 weeks specimen, 4x−10x. **(H)** The V-G staining of hard slice of muscle-reattachment functional prosthesis through cross-section at 8 weeks, 4x. **(I)** The HE staining of hard slice of muscle-reattachment functional prosthesis through cross-section at 8 weeks; and the muscle tissue with new generated vessel can be observed, 4x. **(J)** The histological analysis of the tissue ingrowth percentage in the *in-situ* replaced prosthesis based on HE stanning. The data are presented as the mean ± SD (**p < 0.01). **(K)** The number of capillaries and vessels in optical microscope scanning filed. The data are presented as the mean ± SD (**p < 0.01).

The average passive MIO of the six goats under general anesthesia was 57.38 ± 2.83 mm pre-operatively, and the average passive MIO was 56.47 ± 3.97 mm at 8 weeks after the operation, with no significant difference (*p* > 0.05). A gross view of the six prosthesis specimens showed that muscle grew into the porous area of the prosthesis (Figure 2D). There was no ectopic bone formation either in or outside the muscle attachment. The average maximum avulsion pressure of the LPM from the condyle in the goat was 0.989 ± 0.036 N/mm^2^, and 0.436 ± 0.038 N/mm^2^ of the LPM from the novel designed prosthesis at 8 weeks (*p* < 0.05, Figures 6A–C). The hard tissue slice with HE staining and V-G staining showed that the biomimetic prosthesis formed increasingly good biological integration with the tissue. It is showed that until the 8th week, the tissue penetrated into the muscle function zone and almost formed complete integration with obvious vessel generated (Figures 6D–I). The prosthesis-tissue junction was mainly collagen fiber binding, and the neonatal vessel structure was visible. And according to the HE stanning and histological analysis, the tissue ingrowth percentage increase from 73.28 ± 5.86 to 93.65 ± 3.78 (*p* < 0.01, Figure 6J), and the number of capillaries and vessels increased to 7.29 ± 1.48 per field as well at 8 weeks specimens (*p* < 0.05, Figure 6K). Also, it has to be admitted that there were still some small blank gaps between the edge of the prosthesis and the tissue.

## Discussion

TMJ is the only movable joint of the cranial and maxillofacial region, which dominates the mandibular movement and participates in important functions such as speech and chewing. Artificial TMJ replacement is an effective method for end-stage TMJ diseases. It has been widely used in European and North America. However, the main problem of artificial TMJ prosthesis is limited joint function, especially in laterotrusion and protrusion movement, which is caused by the loss of LPM attachment and functioning (Wojczyńska et al., 2016). Although there have been many modifications based on mechanical changes, few considered muscle reattachment, until one sutured muscle to a porous structure filled with bone marrow in a designed TMJ prosthesis (Mommaerts, 2019). In this study, we proposed a novel design for LPM attachment to prostheses by using 3D printed porous scaffold and test the possibility of only muscle attachment and ingrowth by *in vitro* and *in vivo* experiments.

It is confirmed that titanium and titanium alloys have good biological compatibility with bone tissue and, as such, have been widely employed in surgical internal rigid fixation, implants, and prostheses (Li et al., 2013; Dai et al., 2016; Bosshardt et al., 2017; Ghanaati et al., 2019). The modification of titanium alloy can be benefit to the tissue integration as more elements has been added to the mixture, which can increase the bone growth and integration (Guo et al., 2013; Liu et al., 2015). The development of 3D printing technology has helped to realize advances in porous scaffold manufacture, which provides a favorable environment for the transportation of cells, blood vessels, tissue metabolites products and nutrients. It is reported that a pore size of about 400 μm is beneficial for the growth of bone tissue (Bobyn et al., 1999), and a large pore size is conducive to the penetration and regeneration of blood vessels (Thorson et al., 2019). Porous titanium also has good osteo-conductivity and can achieve biological fixation with bone. The studies above gave us a clue that the Ti porous structure scaffold may also have the potential for soft tissue reconstruction, thus in this study, we tried to use porous Ti scaffold for muscle attachment.

Research on the adhesion between titanium alloys and soft tissues has gained more and more attention in recent years. In the previous studies, researchers found that the fibroblast can form a dense biological integration with the implants and can reach better results with surface modifications as roughed surface (Lee et al., 2015; Rieger et al., 2015). Furthermore, it was also discovered in the craniofacial surgery that after 1 year implantation, the surface of the titanium plate was generally covered with dense, fibrous connective tissue (Armencea et al., 2019). Janseen et al. found the porous titanium is suitable for the soft tissue ingrowth and combination (Janssen et al., 2009). He used a porous titanium mesh to reconstruct a tracheal support placed under the mucosa of rabbit trachea. After 6 weeks, HE staining revealed that the fibrous connective tissue grew into the titanium mesh and that the porous titanium mesh formed a tight integration with the tracheal cartilage. Additionally, Zhao et al. (2008) implanted the porous titanium mesh into the intervertebral muscle of rats. After 8 weeks, the maximum muscle avulsion force from the porous titanium mesh reached to 2.46 N, and the pressure reached to 0.56 ± 0.23 N/mm^2^, which is smaller than that (0.801 N/mm^2^) in our study.

In tendon reconstruction, Reach et al. (2007) showed that the tendon can heal directly with the porous titanium material and reached 90% of the mechanical strength of the control side 6 weeks after fixation with titanium nails and spacers in the achilles tendon reconstruction of beagles, and good kinematic function as walking and running was also acquired. As it was shown in our study, the bio-connection of porous structure and the soft tissue is mainly formed with collagen fibers, thus how to form a prosthesis-tendon-muscle reconstruction and its function may need further research to meet higher requirement.

In this study, we first proposed the hypothesis that muscle can attach to the prosthesis with porous scaffold, then tested the possibility by muscle cells with the porous scaffold *in vitro*. After positive result, we moved to rat muscle implantation and later on condyle reconstruction in goat. The results showed that both myoblast cells and muscle tissues had good biocompatibility with the porous titanium scaffolds. According to the PCR and western blot experiment, the L6 myoblast cells cultured on the scaffold showed better myogenic capability when compared with the common culture group, but less integrin-β_1_ expression because of the culture dish surface treatment. This serves as an important reminder that future improvement of the scaffold design could consider enhancing the muscle adhesion through material modification, which plays a vital important role in the tissue integration and the metal material scaffold. More irons or structure modifications may be tested for cell adhesion improvement. The implantation of a porous titanium scaffold in rat muscle further confirmed that muscle can grow into the pores of the scaffold *in vivo*, and the integration percentage between muscle and the porous titanium scaffold increased along with the implantation time. Our novel prosthesis for goat condyle reconstruction likewise showed the feasibility of muscle attachment to a prosthesis. Muscles with new blood vessels could be observed in the porous titanium scaffold. In the pore, collagen fibers were integrated with the titanium at the edge, while muscle fibers were integrated in the middle of the pore. This being the case, more time and observation are needed to understand whether muscle fibers will change to collagen fibers like muscle tendons after biomechanical movement. Whereas, the tracking force of the muscle from the prosthesis 8 weeks after implantation reached to 0.436 N/mm^2^, which is still insufficient compared with the one of condyle-LPM attachment (0.989 N/mm^2^). Thus, further research is needed to optimize the material structure and improve the force of muscle attachment to the porous scaffold. In the future study, it may be concerned that the promising myogenic iron could be added into the metal materials to promote the cell adhesion and tissue integration as what has been done in the bone research.

In conclusion, our novel TMJ prosthetic provides the possibility of muscle ingrowth and attachment which may improve mandibular movement in the future.

## Data Availability Statement

The original contributions presented in the study are included in the article/supplementary material, further inquiries can be directed to the corresponding author/s.

## Ethics Statement

The animal study was reviewed and approved by the Shanghai Ninth People's Hospital Human Research Ethics Committee.

## Author Contributions

LZ was involved in the processed experiments, acquisition of data, and drafting the manuscript. YZ helped with the molecular biology experiments. YX helped with the prosthesis design and collecting data of the avulsion force. DH contributed to the study protocol design and the surgery of the goats. XL helped with 3D printing technical details. CL helped with *in vivo* experiments. HZ helped draw the novel prosthesis picture. All authors read and approved the final manuscript.

## Conflict of Interest

The authors declare that the research was conducted in the absence of any commercial or financial relationships that could be construed as a potential conflict of interest.
